# Influence of Body Condition on Influenza A Virus Infection in Mallard Ducks: Experimental Infection Data

**DOI:** 10.1371/journal.pone.0022633

**Published:** 2011-08-16

**Authors:** Dustin M. Arsnoe, Hon S. Ip, Jennifer C. Owen

**Affiliations:** 1 Department of Fisheries and Wildlife, Michigan State University, East Lansing, Michigan, United States of America; 2 United States Geological Survey (USGS) National Wildlife Health Center, Madison, Wisconsin, United States of America; 3 Department of Large Animal Clinical Sciences, Michigan State University, East Lansing, Michigan, United States of America; Erasmus Medical Center, The Netherlands

## Abstract

Migrating waterfowl are implicated in the global spread of influenza A viruses (IAVs), and mallards (*Anas platyrhynchos*) are considered a particularly important IAV reservoir. Prevalence of IAV infection in waterfowl peaks during autumn pre-migration staging and then declines as birds reach wintering areas. Migration is energetically costly and birds often experience declines in body condition that may suppress immune function. We assessed how body condition affects susceptibility to infection, viral shedding and antibody production in wild-caught and captive-bred juvenile mallards challenged with low pathogenic avian influenza virus (LPAIV) H5N9. Wild mallards (n = 30) were separated into three experimental groups; each manipulated through food availability to a different condition level (−20%, −10%, and normal ±5% original body condition), and captive-bred mallards (n = 10) were maintained at normal condition. We found that wild mallards in normal condition were more susceptible to LPAIV infection, shed higher peak viral loads and shed viral RNA more frequently compared to birds in poor condition. Antibody production did not differ according to condition. We found that wild mallards did not differ from captive-bred mallards in viral intensity and duration of infection, but they did exhibit lower antibody titers and greater variation in viral load. Our findings suggest that reduced body condition negatively influences waterfowl host competence to LPAIV infection. This observation is contradictory to the recently proposed condition-dependent hypothesis, according to which birds in reduced condition would be more susceptible to IAV infection. The mechanisms responsible for reducing host competency among birds in poor condition remain unknown. Our research indicates body condition may influence the maintenance and spread of LPAIV by migrating waterfowl.

## Introduction

Birds associated with aquatic environments including Anseriformes (particularly ducks, geese, and swans) and Charadriiformes (particularly gulls, terns, and waders) serve as the natural reservoir for influenza A viruses (IAVs) [1]. Among these birds, dabbling ducks within the Genus Anas are recognized as a primary reservoir [2]. Prevalence of IAV infection in dabbling ducks peaks during autumn when immunologically naïve juvenile waterfowl congregate before migrating south [3], [4]. During migration, many of these birds travel long distances and potentially spread low pathogenic avian influenza viruses (LPAIVs) among countries and between continents. Migration is considered one of the most physiologically demanding activities animals undergo and animals vary in their ability to meet the associated energetic challenges. Despite the elevated prevalence of IAV infection during migration, studies have not fully evaluated how natural variation in waterfowl condition influences a bird's ability to serve as a reservoir host for LPAIV.

An understanding of waterfowl host competence during the migratory period is needed to understand how LPAIV is maintained and transmitted. It has long been assumed that waterfowl are asymptomatic carriers of LPAIV and may transmit the virus during migration [1]. However, recent examination of migratory behavior in wild Bewick's swans (*Cygnus columbianus*) found that swans infected with LPAIV exhibited delayed migration, reduced feeding rates, and shorter flight distances compared to uninfected conspecifics [5]. Furthermore, Latorre-Margalef et al. [6] found that migrating mallard ducks (*Anas platyrhynchos*) infected with IAVs had significantly lower body mass than did uninfected birds. These studies concluded that LPAIV infection may incur larger physiological costs to migrating waterfowl than was previously thought. In response, Flint and Franson [7] provide an alternative explanation, suggesting that birds in poorer condition exhibit reduced immune function and are more susceptible to IAV infection (i.e., “condition-dependent hypothesis” [8]. If their hypothesis is correct, host condition could predict susceptibility to infection, and concentration and duration of viral shedding.

Despite the suggested influence of host condition on IAV infection, laboratory experiments have used birds in normal body condition [9]–[13]. These studies have not accurately represented the range of energetic and immunological condition observed in migrating ducks. For example, migrating waterfowl can experience declines in condition due to inclement weather [14] and decreased food availability [15]. Furthermore, decreases in condition have been correlated with immunosuppression [16], [17]. Owen and Moore [17] found that immune function in migrating thrushes (Family Turdidae) was positively related to body condition. Common eiders (*Somateria mollissima*) experienced reduced immune function during periods of mass loss caused by enhanced incubation effort [18]. It remains unclear how natural fluctuations in body condition influence susceptibility and severity of IAV infection.

We tested the condition-dependent hypothesis using wild-caught juvenile mallards experimentally inoculated with LPAIV. Mallards were selected as the focal species because they are the most abundant migratory dabbling duck in North America and Eurasia, and have accounted for more IAV recoveries than any other species of bird [2], [3], [19]. Studies have shown that mallards in normal physiological condition often remain asymptomatic to IAV infection, but shed high concentrations of the virus [9], [12], [13]. We chose to use wild-caught mallards because previous experimental infection studies have used captive-bred mallards [9]–[13], [20], [21], which may not be truly representative of wild mallard host competency. Accordingly, we included a group of captive-bred mallards for comparison and validation of past research.

In this study, we tested the effect of body condition on susceptibility to infection and viral shedding patterns in wild-caught juvenile mallards challenged with LPAIV. We hypothesized food restriction and subsequent reduced body condition will result in (1) increased susceptibility to infection, (2) increased peak viral load and duration of infection, and (3) decreased antibody production. In addition, we compared susceptibility and viral shedding patterns in wild-caught vs. captive-bred juvenile mallards.

## Materials and Methods

### Ethics Statement

Birds were collected under the authority of the Federal Scientific Collecting (permit no. MB194270), and Michigan Department of Natural Resources Scientific Collecting (permit no. SC1386). Bird handling and all experimental procedures were carried out in accordance with the Guide for the Care and Use of Agricultural Animals in Research and Teaching. The protocol was approved by the Institutional Animal Care and Use Committee of Michigan State University (protocol no. 03-09-052-00).

### Animals

Wild mallards were trapped in September 2009 to coincide with increased abundance of staging waterfowl and seasonal peaks in avian influenza prevalence. Trapping sites (n = 5) were located in shallow marshes surrounded by cropland within the lower peninsula of Michigan, USA. Birds were captured using portable swim-in traps [22] and rocket nets at sites previously baited with whole kernel corn.

Mallards were aged in the field as juvenile or adult by examining wing plumage and cloacal characteristics [23]. The average age of juvenile mallards in the Great Lakes region at the time we were capturing birds was 15 weeks [24]. All juvenile birds were immediately transported to a biosafety level 2 animal containment facility (see Housing, below), weighed to the nearest 1.0 g, and measured for length of flattened wing chord (nearest 1.0 mm), head (0.1 mm), and tarsometatarsus (0.1 mm). Upon capture, birds were tested for previous IAV exposure using the MultiS-Screen enzyme-linked immunosorbent assay (ELISA) (see Serologic Assays, below). Seronegative birds were isolated from one another in separate cages to prevent any potential virus transmission within the facility. All birds were retested with the ELISA at 20 days post-capture to ensure no birds had undetected infections at time of capture. Previous research indicates 20 days is adequate time for seroconversion [9]. Thirty seronegative wild mallards (20 males, 10 females) remained in the study, all other birds were released. Once wild mallards were selected, 10 (8 males, 2 females) twelve week-old mallards were purchased from a closed-flock hatchery (Ridgeway Hatcheries Inc., Ohio, USA). Captive-bred birds were processed the same as wild mallards and were negative for previous IAV exposure. Mallards (wild and captive-bred) were not tested for any additional pathogens or parasites.

### Housing

Mallards were kept in the Michigan State University Research Containment Facility. Birds were randomly assigned to three identical biosafety level 2 rooms and individually housed in 10.6 ft^3^ stainless steel rabbit cages at 20°C. Cages were positioned to allow birds to view one another. Room lighting was adjusted weekly to match the current natural photoperiod in Michigan. Each bird was provided normal access to water, grit, and a commercial maintenance food mash (21% crude protein, 2.7% crude fat, 4.75% crude fiber; true metabolizable energy = 2.82 kcal/g). All birds were acclimated for 30 days before the start of the study.

### Body Condition Index

Capture mass for wild juvenile mallards was adjusted by subtracting the estimated mass of remaining crop contents (1–25% = −28.2 g, 26–50% = −44.4 g, 51–75% = −68.9 g, 46–100% = −119.5 g), and 3.0 g were added for every hour a bird was held prior to recording its initial mass [14]. Body condition was estimated for males and females separately using residuals from an ordinary least-squares (OLS) regression of adjusted mass vs. an index of body size [25]. The body size index was developed by performing a principal component analysis using wing chord, head and tarsus length (PROC PRINCOMP, SAS institute 2002). The first principal component (PC1) was then used as an index of body size. Condition scores were calculated individually by dividing a bird's residual from the OLS regression by its predicted mass. The condition index was assumed to represent normal pre-migration staging condition for juvenile mallards in Michigan. Body condition was not estimated for the captive-bred birds; these closed-flock mallards exhibited significant structural differences (i.e. reduced wing size, large head and tarsus lengths), and the sample size (n = 10 birds) was not large enough to produce a unique and reliable condition model.

### Experimental Design

Wild mallards were randomly divided into three treatment groups (n = 10 birds/treatment). Sex and location of capture were stratified among the groups. After birds acclimated 30 days, diet treatments were initiated following protocols from a pilot study conducted in spring 2009 (Arsnoe unpublished data). Mean treatment conditions were manipulated through food availability to relative conditions decided *a priori* 1) poor treatment = −20% body mass, 2) lean treatment = −10% body mass, 3) normal treatment = ±5% body mass. Captive-bred mallards (n = 10) were maintained at normal condition. Reduced conditions were selected to replicate natural (lean) and substantial (poor) decreases in body condition encountered by migrating waterfowl [26], whereas the normal treatment was designed to represent good body condition without being overweight.

Body condition was assessed every five days by weighing birds to the nearest 1.0 g. In addition, we monitored each bird's body reserves by scoring their keel protuberance and breast muscle development on a 0–3 point scale [27]. When all treatment groups reached their desired condition levels, each bird was inoculated with 1.5 mL of 10^6^ PFU/mL LPAIV (H5N9), 1.0 mL intraesophageally and 0.5 mL oropharyngeally. Following inoculation birds were maintained on their treatment diets to keep them at desired conditions. Cloacal and oral swabs were collected the first 3 days post inoculation (dpi) and every 2 days thereafter until 28 dpi. Swabs from individual birds were pooled together in 1.5 mL of brain heart broth with antimicrobial drugs (100X Anti-Anti, 1.0 mL/100 mL brain heart broth), and transported on dry ice to a −80°C freezer. Blood serum was collected from the brachial vein on 14, 21, and 28 dpi for serologic testing. At 28 dpi mallards were euthanized using CO2 asphyxiation, followed by cervical dislocation.

### Virus

The LPAI virus used was A/Northern pintail/California/44221-761/2006 (H5N9), obtained from USGS National Wildlife Health Center, Wisconsin, USA. This strain of IAV was selected as it has been well characterized and serves as a model waterfowl-derived IAV in our laboratory. Virus was propagated by inoculating the allantoic cavity of 9–11 day old embryonated chicken eggs with 200 µL (1∶10 dilution in DMEM media) [28]. Allantoic fluid was harvested after 4 days, centrifuged and stored in 2 mL aliquots at −80°C. Stock virus was titrated using MDCK plaque assays as described by Tobita et al. [29] and infectivity titers were expressed as plaque forming units (PFU) in 140 µl of egg allantoic fluid.

### Matrix Gene RRT-PCR

Swab samples were thawed at 37°C and homogenized by vortexing. RNA extractions were performed using the QIAamp viral RNA mini kit (QIAGEN, QIAGEN Sciences, Maryland, USA) using 140 µl of sample material, according to the manufacturers' instructions. Real-time RT-PCR assays were performed using protocols targeting the matrix (M) gene [30] using the TaqMan One-Step RT-PCR Master Mix (Applied Biosystems, Foster City, CA, USA) on a ABI Prism 7900 Sequence Detection System. We detected the matrix gene of LPAIV H5N9 at 100 nM and 500 nM final concentration, respectively. Two microliters of the final RNA prep were used as template in a 10 µl final reaction volume. Cycle threshold (Ct) values were standardized by setting the baseline to a threshold of 0.028 for all runs. All Ct-values <40 were considered LPAI virus positive.

### Virus Titration

**Figure 1:**
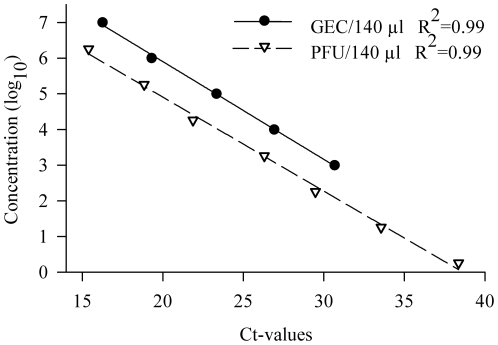
Regression analysis for the calibration of the number of IAV matrix gene copies (circles) and plaque forming units (triangles). The standard curve was generated using a log_10_ dilution series of quantified RNA runoff transcripts or known concentrations of LPAI H5N9 stock virus.

The concentration of LPAIV H5N9 in swab samples was expressed as the number of viral M gene copies or genome equivalent copy numbers (GEC) in 140 µl of swab sample fluid. We calibrated the number of M gene copies by generating a standard curve using a log_10_ dilution series of quantified RNA run-off transcripts as described by Fereidouni et al. [10] (Figure 1). Swab sample titers were extrapolated by entering the observed Ct-value into the standard curve equation.

### IDEXX FlockChek* ELISA

Serum was tested using a commercially available IAV antibody ELISA kit (FlockChek* AI MultiS-Screen, IDEXX Laboratories Inc., Maine, USA). According to the manufacturers' instructions, samples with a signal-to-noise ≤50% were considered positive. Comparison of the FlockChek* ELISA with the more recent NP-ELISA revealed both tests are equally reliable in detecting IAV antibodies [31].

### Hemagglutination Inhibition (HI) Assay

To quantify post inoculation serum antibodies, HI assays were performed using standard protocols [32], using chicken erythrocytes and four hemagglutination units of stock virus used for inoculation. Serum samples were treated with 10% erythrocytes solution to remove nonspecific inhibitors and agglutinins. Samples were processed in duplicate using a 0.5% suspension of chicken erythrocytes. Antibody titers were expressed as the reciprocal of the highest serum dilution yielding complete inhibition of hemagglutination. Samples with HI titers ≥1∶8 were considered positive.

### Data Analysis

The body size index, estimated by PC1, accounted for 60% and 56% of the variance associated with structural measurements (wing chord, head length, and tarsus length) for females and males, respectively. Mean condition scores between treatment groups were compared at the time of capture and when desired condition levels were met using one-way analysis of variance (ANOVA). Post-hoc analyses were carried out using two sample t-tests.

Elevated viral shedding occurred through 5 dpi for most birds. Therefore, analysis of average peak viral load was performed during the first 5 dpi using repeated-measures ANOVA. Mallards were considered infected if they seroconverted post inoculation (either ELISA or HI tests), or shed viral RNA≥3 dpi. In the analysis, samples that did not exhibit detectable viral RNA were assigned a value of zero, and birds considered uninfected were removed from all analyses. Viral M gene copies were transformed using log (base 10).

Shedding duration was calculated for each infected bird as the last day post inoculation where viral RNA was detected. When a bird tested negative for viral RNA on two consecutive sampling events (i.e. minimum 4 days after last positive sample) it was considered no longer shedding virus. In addition, shedding frequency was calculated for each infected mallard as the number of positive samples detected from the 4 sampling days between 1–5 dpi. Shedding duration and frequency data were not normally distributed (Shapiro-Wilk, p<0.05). Therefore, overall group comparisons were done using a nonparametric Kruskal-Wallis ANOVA on ranks, and post-hoc analyses were conducted using Mann-Whitney rank sum test.

Serologic response was compared among treatment groups on 14, 21, and 28 dpi using only infected mallards. For analysis, negative samples (HI titer<8) were assigned a value of half the minimum detectable titer as described by Stephenson et al. [33]. All HI titers were transformed using log (base 2). Analyses of antibody production were conducted using repeated-measures ANOVA. All repeated-measures post-hoc analyses were done using Tukey's HSD (honestly significant difference). The alpha level was set at 0.05 for all analyses, and derived p values correspond with two-tailed tests. Analyses were performed using PASW 18.0 (PASW, 2010).

## Results

**Figure 2:**
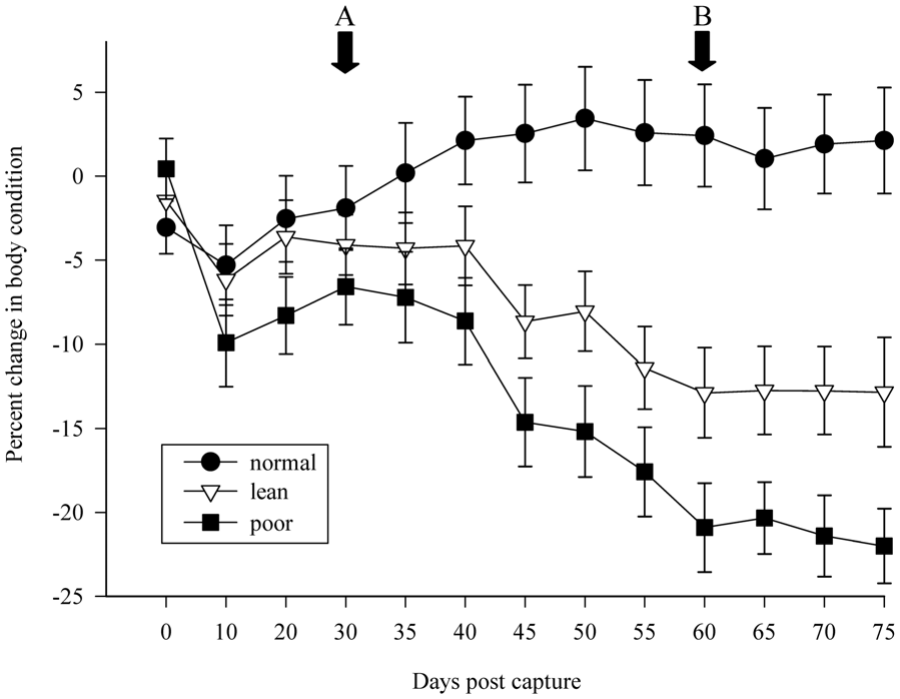
Body condition for wild mallard treatment groups throughout the study. Data points represent mean condition (±1 standard error), A = start of diet manipulation, B = LPAIV H5N9 inoculation.

A total of 81 (43 males; 38 females) juvenile mallards were captured during September 2009. Influenza A virus antibodies were detected in 51% of birds (17 males; 24 females) using ELISA. Of the 40 seronegative mallards available for the study, 10 were released because they did not acclimate to captivity. Mean condition scores for the three wild treatment groups were similar at the time of capture (one-way ANOVA; F_2,27_ = 1.12, p = 0.34; Figure 2). All (wild and captive-bred) birds (n = 40) adjusted well to captivity and were eating and drinking normally at the end of the acclimation period ending on day 30. Food manipulation significantly separated wild treatment group conditions by day 60 (F_2,27_ = 18.0, p = <0.001; Figure 2). Mean condition score for mallards in the poor treatment (−21%) was significantly reduced compared to birds in the lean treatment (−13%) (two-sample t-test; t = 2.13, p = 0.046) and normal treatment (2%) (t = 5.79, p = <0.001). Condition score for lean birds was lower than for mallards in the normal treatment (t = 3.77, p = 0.001). Wild mallards in the normal treatment surpassed their predicted mass during the first five days of diet manipulation, and their mean condition remained elevated (+1.0–2.4%) for the remainder of the study. Body condition of the captive-bred mallards increased by an average of 0.5% during diet manipulation.

### Susceptibility to LPAIV (H5N9) Infection

A total of 37 out of 40 (92.5%) study birds were considered infected (seroconverted or shed detectable viral RNA≥3 dpi) following LPAIV H5N9 inoculation. Two wild birds in the poor treatment and one captive-bred mallard were determined to be uninfected. Body condition scores for the uninfected poor treatment birds at the time of LPAIV challenge were −22% and −24%, whereas the uninfected captive-bred mallard gained 7.4% body mass during diet manipulation. Among all infected mallards, 35 of 37 (94.5%) birds shed detectable viral RNA. The two birds that produced H5 specific antibodies but did not shed detectable viral RNA were wild mallards in the poor treatment and their condition scores were −18% and −36% at the time of LPAIV challenge. Following LPAIV challenge, no birds exhibited clinical signs of disease.

### Average Peak Viral Load

**Figure 3:**
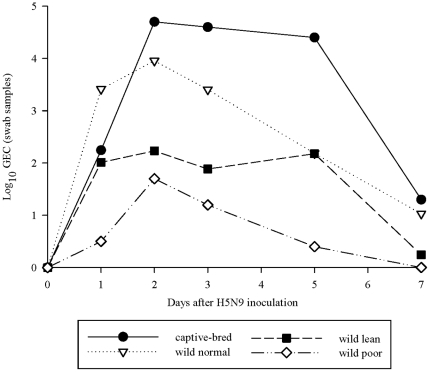
Average viral shedding for captive-bred and wild mallard treatment groups. Data points represent mean genome load (log_10_ GEC/140 µl of swab sample fluid) for each day sampled.

The average peak viral genome load (log_10_ GEC/140 µl of swab sample fluid) for all treatment groups peaked at 2 dpi (wild normal = 3.96; lean = 2.23; poor = 1.70; captive-bred = 4.69) and the bulk of shedding continued through 5 dpi (Figure 3). Virus excretion in wild mallards during the first 5 dpi among treatment groups varied significantly (repeated-measures ANOVA; F_2, 25_ = 5.18, p = 0.013). In general, higher viral genome loads were observed in treatment groups with higher relative condition scores (i.e. greater food availability) (Table 1, Figure 3). Wild mallards in the poor treatment shed less virus than birds in the normal treatment (Tukey's HSD; M = 2.30, p = 0.010) but not significantly less than the lean treatment mallards (M = 1.17, p = 0.213). We were unable to detect a difference in peak shedding concentration between ducks in the normal and lean treatments (M = 1.13, p = 0.274). Wild and captive-bred birds fed normal exhibited similar peak viral loads (F_1, 17_ = 0.79, p = 0.39).

**Table 1:** Group mean daily (±1 SD) viral genome load (log_10_ GEC/140 µl swab sample fluid) for mallards inoculated with LPAIV H5N9.

Treatment group	Days post inoculation				
	1	2	3	5	7
Captive-bred	2.2±1.85	4.7±2.04	4.6±2.66	4.4±1.52	1.3±1.34
*min-max (n)*	*1.6–4.8 (7)*	*2.8–6.1 (8)*	*4.7–7.0 (7)*	*1.0–6.1 (9)*	*1.7–3.3 (5)*
Wild normal	3.4±1.89	4.0±1.8	3.4±2.18	2.2±2.35	1.0±1.44
*min-max (n)*	*1.3–5.7 (9)*	*1.6–6.6 (10)*	*1.9–6.0 (8)*	*3.5–5.2 (5)*	*1.7–3.4 (4)*
Wild lean	2.0±1.39	2.2±1.64	1.9±1.98	2.2±2.36	0.2±0.76
*min-max (n)*	*0.8–4.4 (8)*	*1.7–5.9 (8)*	*1.0–6.4 (7)*	*1.2–6.9 (6)*	*2.4 (1)*
Wild poor	0.5±0.92	1.7±2.46	1.18±2.04	0.4±1.13	-
*min-max (n)*	*1.6–2.3 (2)*	*1.9–7.2 (4)*	*1.8–5.8 (3)*	*3.2 (1)*	-

Mean genome load is calculated using only infected mallards (birds that seroconverted or shed detectable viral RNA≥3 dpi). Minimum and maximum values are reported for the number of birds *(n)* with detectable viral RNA on the given day.

**Figure 4:**
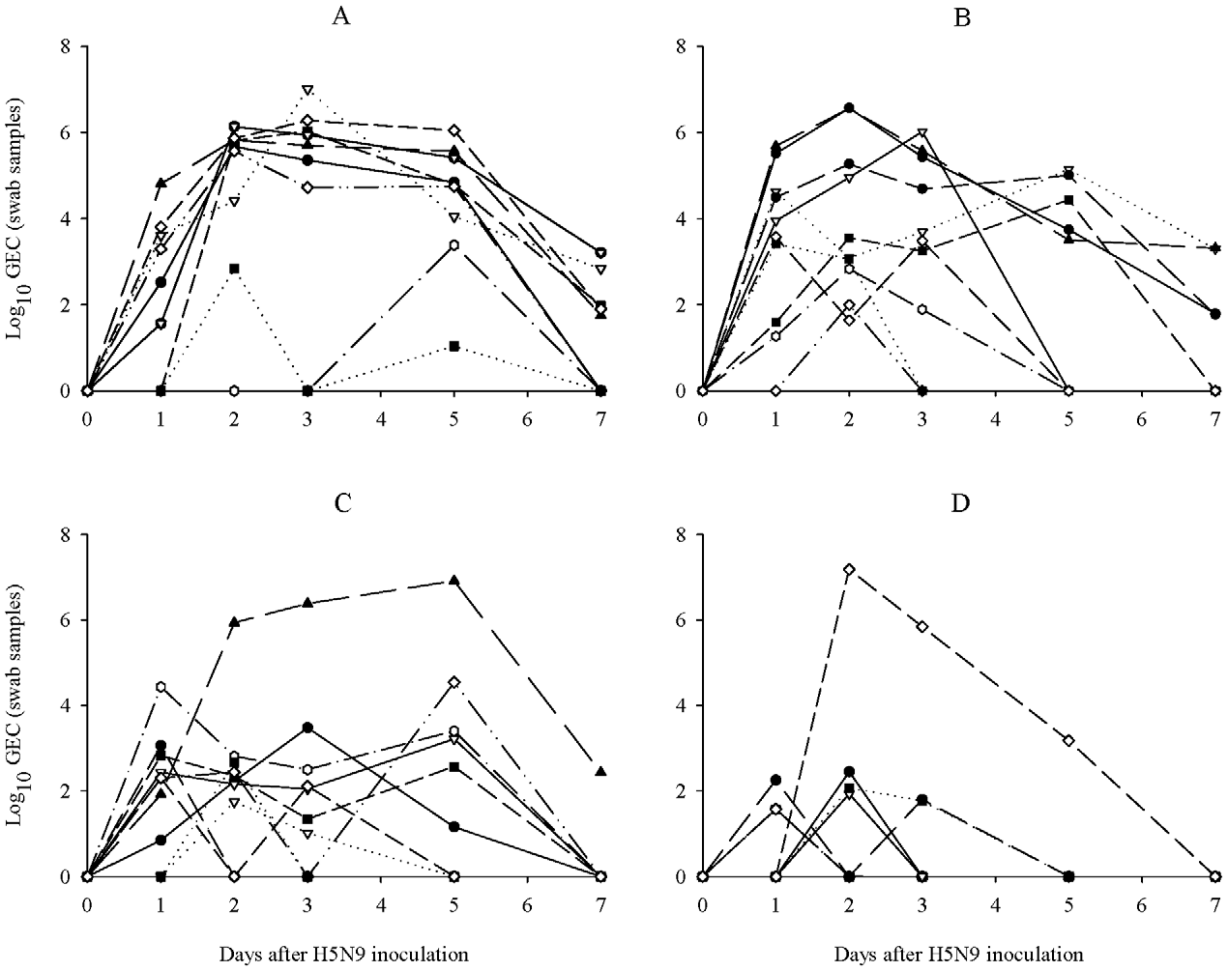
Viral shedding profiles (log_10_ GEC/140 µl of swab sample fluid) for all infected mallards with detectable viral RNA using matrix gene RRT-PCR. A: captive-bred (n = 9), B: wild normal (n = 10), C: wild lean (n = 10), D: wild poor (n = 6).

Concentration of viral shedding was highly variable within treatment groups during the first five days of infection (Figure 4). Average genome loads and standard errors are presented for groups in Table 1. Inspection of individual shedding patterns found a total of 14 mallards that excreted viral RNA at high concentration (≥5.0 log_10_ GEC/140 µl). The majority of these birds were wild and captive-bred mallards fed normal (wild, n = 5; captive-bred, n = 7), whereas only two birds came from reduced condition treatments (lean, n = 1; poor, n = 1). However, peak genome load in the above mentioned poor bird (7.2 log_10_ GEC/140 µl) and lean bird (6.9 log_10_ GEC/140 µl) were the highest observed across all wild mallards.

### Duration of Viral Shedding

**Figure 5:**
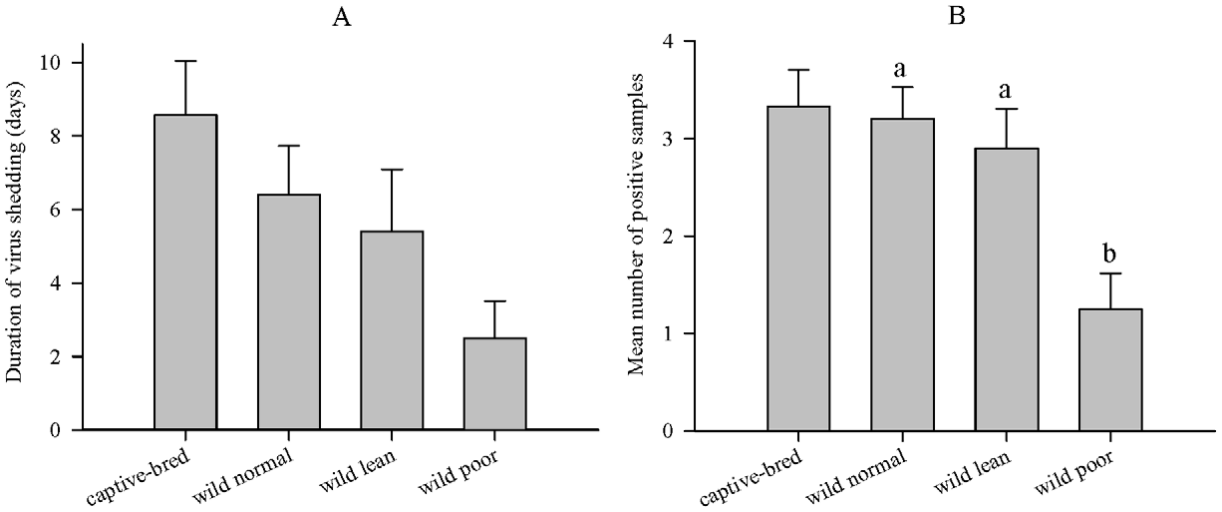
Duration of viral shedding (A) and mean number of positive samples (B) for captive-bred and wild mallard treatment groups. Bars represent means ±1 standard error, letters identify significant differences among wild mallard treatment groups (Mann-Whitney rank sum, alpha = 0.05). The mean number of positive samples was calculated from the 4 sampling days between 1–5 dpi.

Duration of viral shedding among infected birds ranged from 1–20 dpi. Among wild mallards, mean duration (days) of shedding was largest in groups with higher condition scores (wild normal = 6.4; lean = 5.4; poor = 2.5) (Figure 5). Intermittent shedding beyond 5 dpi was more common in wild mallards fed normal (n = 5), whereas only two birds in reduced condition treatments (lean, n = 1; poor, n = 1) shed virus past 5 dpi. Despite these relationships, we were unable to detect a significant difference in shedding duration among wild mallards (Kruskal-Wallis ANOVA; H = 5.86, 2 d.f., p = 0.053). In addition, mean duration of shedding in captive-bred birds (8.6 days) was similar to wild birds fed normal (H = 1.43, 1 d.f., p = 0.23).

The number of positive samples through 5 dpi differed among wild mallard groups (H = 9.67, 2 d.f., p = 0.008; Figure 5). Mean number of positive samples in poor mallards (1.25) was significantly less than for birds fed normal (3.2) (Mann-Whitney rank sum; U = 8.0, p = 0.004), and for lean mallards (2.9) (U = 13.5, p = 0.018). No differences were found between wild normal and lean treatments (U = 45.0, p = 0.71), or between wild and captive-bred birds fed normal (H = 0.253, 1 d.f., p = 0.62).

### Serologic Response

After LPAIV H5N9 challenge, 33 out of 40 (82.5%) study birds tested seropositive using ELISA, whereas 32 out of 40 (80%) had detectable levels of H5-specific antibodies according to HI tests. Overall antibody production at 14, 21, and 28 dpi was similar across wild mallard treatments (repeated-measures ANOVA; F_2, 25_ = 1.28, p = 0.30; Table 2), but differed between wild and captive-bred birds fed normal (F_1, 17_ = 4.51, p = 0.049). Mean HI titer for infected captive-bred birds was higher than wild birds in the normal treatment on all days sampled (Table 2).

**Table 2:** Serological status (mean ± SD) of mallards before and after LPAIV H5N9 challenge using ELISA and HI tests.

Treatment group	ELISA^1^				H5N9 HI^2^			
	B.I.^3^	14*	21	28	B.I.	14	21	28
Captive-bred	0.93±0.09^4^	0.32±0.19	0.38±0.24	0.38±0.25	< 3	4.9±2.03	4.3±1.66	2.8±1.62
Seropositive *(n)*	*(0)*	*(7)*	*(6)*	*(6)*	*(0)*	*(7)*	*(7)*	*(7)*
Wild normal	0.87±0.15	0.30±0.19	0.36±0.23	0.37±0.23	< 3	3.4±1.34	3.2±0.92	2.8±0.63
Seropositive *(n)*	*(0)*	*(9)*	*(6)*	*(6)*	*(0)*	*(7)*	*(8)*	*(7)*
Wild lean	0.86±0.12	0.21±0.10	0.33±0.26	0.36±0.26	< 3	3.2±0.79	3.0±0.82	2.2±1.32
Seropositive *(n)*	*(0)*	*(10)*	*(8)*	*(8)*	*(0)*	*(8)*	*(8)*	*(5)*
Wild poor	0.84±0.10	0.28±0.27	0.42±0.27	0.43±0.24	< 3	3.4±1.19	3.6±1.92	3.4±0.92
Seropositive *(n)*	*(0)*	*(7)*	*(6)*	*(6)*	*(0)*	*(6)*	*(5)*	*(6)*

* Bold numbers indicate days after LPAIV H5N9 inoculation.

1 The ELISA scores represent the signal to noise (S/N) ratio where values ≤0.50 are considered seropositive.

2 The hemagglutination inhibition (HI) values represent the mean titer (log_2_) of sera samples.

3 Before LPAIV H5N9 inoculation.

4 Mean (±1 SD) test scores include all infected birds in each treatment.

## Discussion

Our study demonstrates body condition significantly influences susceptibility to infection and viral shedding patterns in wild-caught juvenile mallards challenged with LPAIV H5N9. However, our findings were contrary to our original predictions based on the condition-dependent hypothesis in which birds in poor condition would experience reduced immune function and increased susceptibility to infection [7]. Here we find birds in normal condition were more susceptible to LPAIV infection, shed higher peak viral loads, and shed viral RNA more frequently compared to birds in reduced condition. A clear trend was observed among wild birds showing a positive relationship between body condition and host competence.

Previously, it has been assumed LPAIV infections remain asymptomatic in wild waterfowl with little or no effects on life-history parameters. However, recently van Gils et al. [5] determined LPAIV infection decreased migratory performance in Bewick's swans, and Latorre-Margalef et al. [6] found mallards infected with IAV were leaner than uninfected conspecifics. The latter study has generated ongoing debate on whether IAV infection influences body condition of migrating waterfowl, or vice versa [7], [8]. Both sides acknowledge the possibility that birds may become immunosuppressed during migration due to reduced energy stores, and therefore suggest further studies are needed to conclusively discriminate between these two hypotheses. To our knowledge, our study is the first to evaluate the condition-dependent hypothesis using IAV infected waterfowl. We have provided evidence that body condition influences IAV infection in wild juvenile mallards, however, the mechanisms responsible for our findings remain unclear and contrary to those suggested by the condition-dependent hypothesis [7].

**Figure 6:**
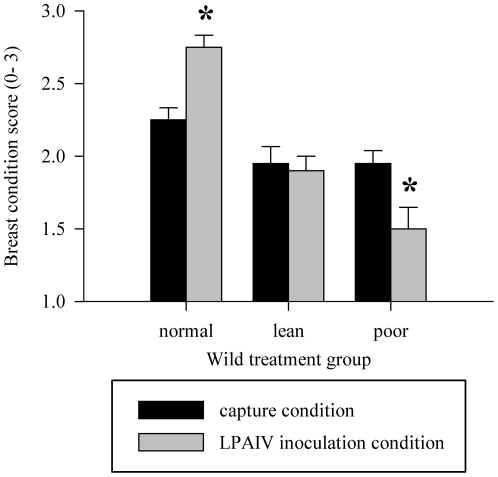
Breast condition score for wild mallard treatment groups at capture vs. LPAIV H5N9 challenge. Bars represent mean condition score ±1 standard error (n = 10), asterisks represent significant within group differences (paired t-test, alpha = 0.05).

Most research examining host nutrition and susceptibility to infectious disease provides overwhelming support for our original hypotheses. In general, studies have found malnutrition increases susceptibility and severity of infection with most microbial agents [34]. In the case of IAV infection, deficiencies in vitamins A and C, selenium, and protein have increased susceptibility and burden of disease [35]–[39]. These findings were attributed to reduced immune function caused by limitations in one or several essential nutrients, vitamins, and/or dietary protein [38], [40]. If we assume mallards in poor condition were malnourished, as indicated by significant decrease in breast condition score (Figure 6), then our findings contradict the well established trend described above. Therefore, we propose the relationship between body condition and LPAIV infection in waterfowl is more complex than previously thought.

Review of previous research, however, outlines three additional factors that may influence the observed relationship: (1) duration of food restriction, (2) depletion of subcutaneous fat reserves, and (3) changes in intestinal composition due to reduced food intake. Past studies have shown certain conditions of malnutrition increase hosts resistance to viral infection [41], [42]. Sprunt and Flanigan [41] found mice and chickens fed reduced protein diets exhibited a cyclic pattern of susceptibility relative to those fed high protein diets. Protein restriction increased mice and chickens' susceptibility for the first two weeks, decreased their susceptibility from three to six weeks, and then beyond seven weeks increased their susceptibility again. Susceptibility to infection was lower when fat reserves had been depleted and the animals had initiated catabolism of available protein reserves. Likewise, diets high in protein have been correlated with increased resistance to viral infection [40]. Our findings support those of Sprunt and Flanigan [41], as mallards in poor condition were less susceptible to infection after four weeks of food restriction. Furthermore, birds in poor condition had significantly reduced keel scores when challenged with LPAIV (Figure 6); thereby indicating these mallards were emaciated and may have transitioned to catabolism of protein reserves [27]. Future studies may examine susceptibility to IAV infection during shorter periods of food restriction (<4 weeks) to see if the relationship continues to support the pattern observed by Sprunt and Flanigan [41].

Changes in intestinal composition from reduced food availability may be responsible for decreased susceptibility and viral shedding among mallards in reduced condition. Food deprivation has been shown to reduce the relative amount of mucin glycoprotein in the intestinal tract. Smirnov et al. [43] examined intestinal mucin in chickens fasted for 72 hours, and found acute food deprivation decreased mucin thickness throughout the small intestine. In rats deprived 50% of their daily intake for five weeks, the concentration of intestinal mucin was significantly reduced compared to control animals fed normally [44]. In waterfowl, LPAI viruses preferentially bind to sialic acid (SA) receptors which occupy terminal positions on mucin glycoproteins within the intestinal tract [45], [46]. Therefore, it is plausible that decreased abundance of mucin may reduce SA expression and inhibit viral attachment and propagation. While we did not investigate this possible mechanism, concerning reduced food availability, intestinal mucin, and SA expression for differences in resistance, it is potentially an important determinant that warrants further investigation.

Our serological data from both ELISA and HI tests indicate reduced body condition does not affect mallard antibody production in response to LPAIV H5N9 challenge. These findings do not support our initial prediction in which resource limited birds experience decreased antibody production [18], [47]. It is well understood that maintaining and using the immune system is energetically costly [48]. During periods of limited food access, Buehler et al. [16] found migrating shorebirds suppress more costly acute-phase immune responses (phagocytosis, fever, inflammation) in order to maintain a baseline level of immune function. Thus, mallards fed restricted diets may have down regulated some components of immune function and retained the ability to produce specific antibodies. Alternatively, mallards in reduced condition may have adequate resources to enable production of a low-level humoral response typical of LPAIV infections [11], [45]. However, it is possible that we missed a difference in antibody production immediately following seroconversion by not sampling birds earlier than 14 dpi; in previous studies mallards have seroconverted as early as 7 dpi [10].

Factors other than host body condition are known to influence the epidemiology of IAV infection in migrating waterfowl. Immunity induced by prior IAV exposure has been shown to reduce susceptibility and viral shedding by waterfowl during subsequent infections [10], [11], [45]. For example, Jourdain et al. [11] demonstrated LPAIV H5N7 infection in juvenile mallards reduced viral RNA excretion during homosubtypic reinfection, and protected some birds against heterosubtypic LPAIV H5N2 reinfection. Similar repeated IAV exposures are thought to induce transient immunity in migrating waterfowl, which provides one explanation for the low IAV infection prevalence (<1%) among wintering waterfowl [6], [11]. Alternatively, species migratory behavior may also play a role in prevalence of IAV infection. Blue-winged teal (*Anas discors*) are early migrants that typically leave the staging grounds prior to the seasonal peak in IAV infection prevalence. As a result, they remain susceptible and have exhibited IAV prevalence rates ≥30% on the wintering grounds [49]. It is clear these and other factors influence the maintenance and spread of LPAI viruses by migrating waterfowl, and our findings suggest host body condition may also play an important role.

In summary, we have taken an important step in showing how host body condition may play a significant role in the epidemiology of LPAIV infection in mallards, and presumable other waterfowl species. We provide evidence that (1) mallards in poor condition are less susceptible to infection compared to birds in normal condition, (2) concentration and duration of viral shedding are positively correlated with host condition, and (3) body condition does not affect mallard specific antibody response following LPAIV challenge. The study also suggests that captive-bred mallards may replace wild mallards in future experimental models where birds are maintained at normal condition. The precise mechanisms of decreased host competence among mallards in reduced condition remains unknown. If susceptibility follows a cyclic pattern, as indicated by Sprunt and Flanigan [41], then birds would first encounter a period of enhanced virus transmission in response to food deprivation. Additional field and laboratory studies under varying durations of food restriction are encouraged to clarify this proposed relationship. Furthermore, studies should evaluate the influence of body condition using different LPAIV subtypes. Such data would help explain how body condition influences waterfowl host competency during LPAIV infection, and improve future LPAIV transmission models.
